# The Implementation of Restorative Care and Factors Associated with Resident Outcomes in Long-Term Care Facilities in Taiwan

**DOI:** 10.3390/ijerph16203860

**Published:** 2019-10-12

**Authors:** Yu-Hua Wang, Li-Fan Liu, Ling-Hui Chang, Chien-Hsin Yeh

**Affiliations:** 1 Institute of Gerontology, National Cheng Kung University, Tainan 701, Taiwan; m6cj841881@gmail.com; 2 Department of Occupational Therapy, National Cheng Kung University, Tainan 701, Taiwan; lhchang@mail.ncku.edu.tw; 3 Yung Shin Social Welfare Foundation, Taichung 437, Taiwan; u90141@sunrise.hk.edu.tw

**Keywords:** long-term care, facility, restorative care, effectiveness

## Abstract

This study aimed to analyze how restorative care is implemented in long-term care facilities and factors associated with resident outcomes in Taiwan. A one-group pre-test and post-test design was adopted in 24 long-term care facilities by collecting a sample of 310 participants at the baseline and 210 at six months. Participants were residents aged 65 or over, and were being constrained, used diapers, or were bedridden, or a combination of these. Their physical and mental functions were measured using Activities of Daily Living (ADLs), Instrumental Activities of Daily Living (IADLs), Mini-Mental State Examination (MMSE), Geriatric Depression Scale-15 (GDS-15), and EuroQol-5D (EQ-5D). Mean differences in the outcomes were analyzed, and mixed effect models were used to examine influencing factors. The results showed that most of the participants had good family support. However, participants with better family support were more likely to drop out. Improvements were found in the residents’ outcomes on physical function, depression and quality of life. Social support was a significant influencing factor on most of the outcomes. In conclusion, restorative care was found to have positive effects on residents’ physical function and helped maintain mental function. Sufficient support and communication between participants, families, and staff in facilities are key factors leading to positive outcomes.

## 1. Introduction

### 1.1. Challenges Faced by Older People with Disabilities

Aging causes inevitable functional decline and disabilities. The deterioration of biological and psychosocial functions renders older people particularly vulnerable to the loss of independence, dignity, and privacy, which may cause them to struggle to maintain their homes, self-worth, and dignity in later life.

Things have gotten worse when it comes to institutional settings. Residents often receive inappropriate care, which forces them to become accustomed to relying on poor caregivers due to various factors, including management and manpower, in turn leading to the accelerated deterioration of patients. Taking the use of physical restraints in institutional care for example, the use ratio in Taiwan is approximately 25% to 54%, and it is likely proportional to the length of stay [1]. However, in terms of human rights and quality of care in long-term care (LTC) facilities, the consensus should be to safeguard the dignity and self-reliance of the elderly, therefore assisting them to continue their previous roles and lead fulfilling lives [2].

### 1.2. Independent Living, Restorative Care, and Its Theoretical Perspectives

The independent living (IL) movement initiated by people with disabilities began in the 1960s. The movement is more than an effort to acquire new rights and entitlements; it has spawned a new service delivery model [3]. From the perspective of person-environment interaction, disability should always be considered to be a dynamic process that requires coping with environmental barriers, where the disabled person must shed the patient or client role for the consumer role [4].

In the last three decades, gerontological research has integrated similar ideals and developed models or hypotheses to interpret individual scenarios and possible solutions—both personal and contextual factors should be considered and adjusted to create a subjectively independent individual, so that people can take part in preferred activities on their own initiative.

In 1987, the Omnibus Budget Reconciliation Act (OBRA) in the USA mandated that residents of LTC facilities attain and maintain the highest level of function in activities of daily living (ADLs) possible unless the circumstances of the individual’s clinical condition demonstrate that decline is unavoidable. Since then, the LTC industry has struggled to develop and implement effective restorative care programs [5,6,7]. Restorative care activities will, for example, involve walking to the dining room for some residents rather than going in a wheelchair. Alternatively, restorative care might include hand bathing to encourage passive range of motion. As defined by Atchinson (1992), restorative care aims to maximize a resident’s abilities, focuses on what a resident can do, improves self-image and self-esteem, reduces the level of care required, and eliminates or minimizes the degrading features of LTC, such as restraints, incontinence, and supervised feeding [8]. Medicare in the US provides some restorative care coverage if the resident is provided with two or more nursing rehabilitation activities for 15 or more minutes a day for six or more consecutive days [9].

In terms of theoretical perspectives, the Social Ecological Model (SEM), which addresses intrapersonal, interpersonal, environmental, and policy factors, provides a framework for facilitating changes in the current nursing philosophy in institutional settings using a multilevel perspective [7]. At the intra-individual level, a number of factors that lead to functional limitations, such as cognitive impairment, comorbid and acute medical problems, and depression, have been addressed [5]. At the inter-personal level, the theory of self-efficacy (SE) [10] has been used to guide intervention with the intent of strengthening SE and outcome expectations (OEs) among residents and nursing assistants (NAs), thereby increasing the likelihood that NAs will implement restorative care and that residents will engage in functional tasks and physical activities.

Environments that facilitate physical activities can reduce functional decline and enable people to achieve their highest level of function and well-being [11,12]. Policy-related issues at the facility and/or national level are critical to consider since inappropriate policies may serve as barriers to both function and physical activity.

### 1.3. Restorative Care Intervention: from Japan to Taiwan

Japan introduced a similar concept called “self-supporting” care when LTC insurance was introduced in 2000. According to Professor Takeuchi’s care theory, the four specific elements related to implementing self-supporting care are water (1500 cc per day), diet (1500 calories per day), exercise (walking for 30 min or 2 km per day, or POWER rehabilitation), and excretion (regular voluntary excretion at least once every 3 days), while adhering to three principles: remaining non-bedridden, not using diapers, and not being constrained [13].

The Japanese model of self-supporting care was introduced to Taiwan in 2006. Self-supporting care does not necessarily mean that an elderly person can live independently, but rather that they can live while only receiving necessary care from others that compensates for unavoidable functional losses. Therefore, in addition to the physical level, a process of communication and exchange that reveals the interests, ideas, and goals of the elderly is also emphasized to ensure that participation in the program is voluntary and further promotes spiritual satisfaction as well as quality of life [14].

In the American and Japanese models mentioned above, there is a newly rising care philosophy with different terms used from country to country that shares the same principles. It is known as ‘restorative care’ in the USA, Australia, and New Zealand, and ‘reablement’ in the UK, Ireland, and Denmark [15,16]. When the care philosophy is implemented at various sites, such as in the home or in a care facility, it derives the respective characteristics and we used the term as restorative care while the main implemented site examined is LTC institutions in Taiwan.

### 1.4. Outcomes of the Restorative Care Approach

Several studies have noted some positive outcomes associated with the implementation of the restorative care approach. Outcomes have generally been focused on ADLs, physical activity, or psychosocial factors such as mood or levels of anxiety. These studies demonstrated the benefits of restorative care in decreasing caregiver burden, improving resident functional abilities, decreasing pain, anxiety, and disruptive behavior, and increasing the time and amount of participation in physical activities. Additional outcomes such as quality of life, gait, falls, grooming, motivational factors such as SE, and OEs remained unchanged [17,18]. There is no evidence that restorative care increases fall or other adverse events [19].

The effectiveness of the Japanese model has not yet been fully documented since it was introduced in institutional settings in 2006 and developed vigorously in Taiwan. To the best of our knowledge, there is rare academic research incorporating scientific methods that have analyzed the implementation and outcomes of the restorative care program in Taiwan, but evidence from case reports might not convince frontline staff [20]. Therefore, this study aimed to describe and analyze how restorative care is implemented in LTC facilities and to explore its effect on resident outcomes and associated factors in order to generate evidence and provide practical suggestions for this care philosophy based on the results and to motivate further research.

## 2. Materials and Methods

The aim of this study is to describe and analyze how restorative care is implemented in LTC facilities and to explore residents’ outcomes and factors associated with service delivery and the development of restorative care, which will generate evidence to support commissioners and practitioners as they make decisions about the organization. This study was approved by the Institutional Review Board of Ethics at National Cheng Kung University Hospital (no. A-ER-105-435), Taiwan.

### 2.1. Study Design

This study analyzed secondary data collected from a Non-Profit Organization foundation in central Taiwan that hosted a promotion project of restorative care. A single group pre- and post-test design was used with measurements taken at the baseline (T0) and 6 months (T1) after implementation of the restorative care intervention. Residents were recruited from 24 LTC facilities across Taiwan that had joined the project.

### 2.2. Facilities and Samples

All the LTC facilities interested in this project were recruited by the foundation for a restorative care implementation program in 2017–2019—managers and the primary care staff of each facility had joined workshops and received training before initiating the program, and supervisors from the foundation conducted regular meetings with them every month during the intervention period. A total of 24 facilities participated in the program: 6 of the facilities were located in northern Taiwan, 7 were in central Taiwan, 10 were in southern Taiwan, and 1 was in eastern Taiwan. Regarding sampling, all of the LTC facilities recruited residents using consecutive sampling. Residents living in the study sites were approached for eligibility screening and were included in the program when they signed the informed consent form. The residents were eligible to participate in the program if they had a life expectancy greater than 6 months and met at least one of the following conditions: were constrained, used diapers, were intubated with endotracheal tube/Foley catheter/nasogastric tube, were unable to move around without a wheelchair, or were able to stand for 5 s with or without an assistive device.

### 2.3. Measurements

Outcome measures for the study focused on the intervention and resident’s participation in restorative care, the social support of participants, and the influence on their physical and mental functions such as generic outcome measures of the ADLs, Instrumental Activities of Daily Living (IADLs), Mini-Mental State Examination (MMSE) and Geriatric Depression Scale-15 (GDS-15). The following measures were recorded at the baseline and 6 month follow up by resident’s primary caregivers—mainly the certified care assistants in facilities, who were familiar with them. Primary caregivers/family members were regarded as a proxy to answer some questions for the participants if the participants had communication problems or could not understand the questions.

#### 2.3.1. Intervention Notes

This is a form created by experts of restorative care invited by the foundation for recording care plans instructed by restorative care supervisors, as well as resident’s demographic information, including the name of the facility, case identity, gender, disease, family support, medicine use, long-term goal, etc. Details of the supervisors’ instructions were described in the care plan, including water intake, meal types, diaper types in the daytime and nighttime, standing/walking ability, seat situation when dining, constraint type and time, etc. The note was reviewed and revised every 2 months when supervisors from the foundation conducted a regular meeting, and they would confirm the residents’ status in person to ensure the consensus of the care plan with primary caregivers. These notes were recorded 4 times in the study process, and they mainly recorded the process indicators of the restorative care program.

#### 2.3.2. The Barthel Index

This is a measure of function that specifically assesses a patient’s performance of activities of daily living (ADLs), commonly used in Taiwan’s LTC system [21]. There is sufficient evidence for the reliability and validity of the Barthel Index when used with older adults [22].

#### 2.3.3. The Lawton Instrumental Activities of Daily Living (IADLs)

This scale is mainly for assessing daily activities that are more complex and require higher ability than basic ADLs [23]. Since the study area was LTC facilities, we modified the index to include 6 items, namely cooking, housekeeping, shopping, going out, using the phone, and taking medicines. The scoring refers to the need assessment instrument in LTC issued by the central government in 2007. If a resident needs assistance in more than 3 items, they were considered disabled in performing IADLs.

#### 2.3.4. Mini-Mental State Examination (MMSE)

The evaluation contains components such as orientation, attention, memory, language, oral comprehension, behavioral ability, and constructive ability [24]. The total score is 30. In institutional care, a score in the range of 0–15 indicates severe cognitive impairment, 16–23 indicates mild cognitive impairment, and 24–30 indicates normal cognitive ability.

#### 2.3.5. The Geriatric Depression Scale-15 (GDS-15)

The scale compiled by Yesavage et al. was edited by domestic scholars through empirical research to make the questions easy to answer for the elderly [25]. A score of 0–5 indicates good condition, 6–9 indicates melancholy, and scores higher than 10 indicate depression (recommended to refer to counseling) [26].

#### 2.3.6. EuroQol-5D (EQ-5D)

EQ-5D is designed for self-completion by respondents, providing a simple, generic measure of health for clinical and economic appraisal. EQ-5D consists of two pages: the descriptive system and the visual analogue scale (VAS). The descriptive system comprises the following five dimensions: mobility, self-care, usual activities, pain/discomfort, and anxiety/depression. Each dimension has three levels: no problems, some problems, extreme problems. The VAS records the quantitative measure of respondent’s self-rated health outcome on a vertical, visual analogue scale.

#### 2.3.7. Interview Schedule for Social Interaction (ISSI)

The Interview Schedule for Social Interaction for institutional elderly is modified from Barrera, Slander, and Ramsay’s (1981) Inventory of Socially Supportive Behavior, with 10 questions reserved for social support networks and society. The domains of the scale are divided into emotional support, information support, social integration, and substantive support, using a 3-point Likert scale for scoring. The higher the score, the higher the satisfaction of social support the participant feels [27].

A summary of the operation definitions of the measurement variables mentioned above is given in Table 1. These variables are used in the repeated measures in the analyses of the mixed-effect models.

### 2.4. Analysis

**Figure 1:**
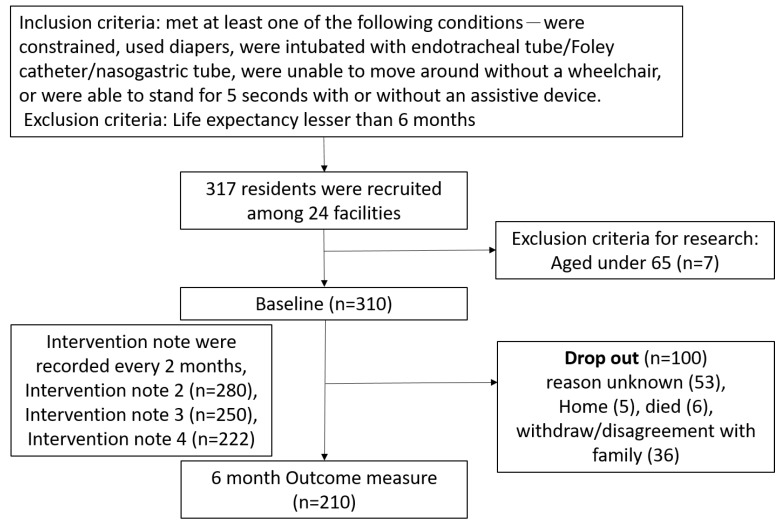
Flow chart of sample creation and attrition.

After the data cleaning process, there were 317 residents participating in the program with valid questionnaires, but 7 of them were ineligible for research criteria as they were under 65 years old. Therefore, we included 310 residents’ data at the baseline. During the course of the 6 month follow-up, 100 (32.25%) participants dropped out, and therefore 210 residents’ data were available for outcome measure. A flow chart of sample creation and attrition is shown in Figure 1.

Descriptive statistics were used to describe resident characteristics. Repeated-measures analysis of variance (ANOVA) was used to identify the changes in items in the intervention note every 2 months. The independent t-test was used to compare baseline differences between residents who finished and dropped out from the restorative care intervention. The paired t-test was used to examine the restorative care’s effect on physical and mental functions over 6 months in residents who remained in the program.

The mixed-effect models were used as inferential statistics to examine the influencing factors on health outcomes of participants in restorative care while controlling for potential confounding of the demographic characteristics of participants and facility variables. The variables regarding participants included age, gender, recruitment conditions, initial health and cognition status, family support (family visit frequency), social support from facility staff (ISSI), which were obtained at the baseline (T0), and the intervention type; these variables were used as static predictors.

Data analysis was conducted using SPSS 20.0 (SPSS, Inc., Chicago, IL, USA) and SAS 9.4 (SAS Institute Inc., Cary, NC, USA). Differences were considered significant at the *p* < 0.05 level of significance.

## 3. Results

### 3.1. Baseline Characteristics

The demographic and health characteristics of the 310 residents enrolled at the baseline are shown in Table 2. The average age was 81.27 years, with males accounting for 40.6% of the total, and the main health conditions were non-stroke neurological diseases (26%) and stroke (20%).

The family visit frequency was high (42.5%) and social support from facility staff was high (ISSI mean score 24.66). Nearly two-thirds of the residents were unable to move around without a wheelchair (63.4%), 77.3% were able to stand for 5 s with or without an assistive device, 74.4% used diapers, 12.9% were constrained, and 6.1% were intubated.

The average physical function was moderate to severe dependency in ADLs (mean score 50.74) and disabled in IADLs (mean score 0.75). The mean MMSE score was 15.56. The residents had a mean utility value of 0.75 in EQ-5D and a VAS score of 59.32. The mean GDS-15 score was 5.03.

### 3.2. Content and Change of Intervention Strategies

The common strategies supervisors used after discussion with facility staff and the resident in person were determined from the intervention notes. The intervention notes were reviewed and recorded at each supervision process every two months. They are summarized as 16 items: exercise/activity training, bed mobility, transfer (bed/chair), sit to stand, mobility training, pain management, fall prevention, assistive device, unconstrained, excretion-related, communication process, self-care bathing, self-care dressing, sleep adjustment, diet/water, and social interaction.

It was found that in each intervention period, the top five strategies were the same, namely sit to stand, mobility training, exercise/activity training, diet/water, and excretion-related strategies. These five strategies are all related to the four elements of Japanese restorative care (water, diet, exercise, and excretion), and were identified as the primary focus in the first 6 months of intervention in our study, especially the exercise element since three of the five top strategies were related to physical activities, and the proportion of exercise/activity training significantly increased in the later period of intervention.

The sit to stand strategy includes static sitting or standing balance, and the dynamic process from sit to stand training. The mobility training strategy includes short/long distance mobilization or gait training. Individualized plans such as resistance band, exercise group, upper/lower body strength/endurance training are categorized to exercise/activity training strategies. As for the diet/water strategy, it contains every approach to motivate or encourage participants to intake enough water and nutrition actively. This may be done through altering meal type, sitting in a chair rather than wheelchair, which results in an inappropriate position to keep trunk balanced while eating, providing flavored water and oral function exercise. Excretion-related strategy includes altering diaper type, removing the diaper, bowel or bladder training and independence in the procedure.

The observed performance corresponds to the top five strategies (focusing on the element of water, diet, exercise, and excretion)was also recorded in intervention note items. As Table 3 shows, these items did show significant improvement along with the program.

### 3.3. Characteristics of Participants Who Had Dropped Out

During the average 6 month follow-up period, there were 100 cases with no post-test information due to personal, family, or institutional reasons.

Since this study is aimed at analyzing how restorative care is implemented in long-term care facilities and factors associated with resident outcomes in Taiwan, the characteristics of and reasons for dropout could be important information. Table 4 shows the differences in the demographic variables and the pre-test scores between the completed group (210 cases) and the drop out group (100 cases) analyzed using the independent t-test.

The drop out group only stayed in the program for an average of 4.68 weeks, and they had lower scores in the Barthel index (*p* < 0.05), EQ-5D (*p* < 0.01), and ISSI (*p* < 0.001), higher scores in GDS-15 (*p* < 0.001), a lower proportion of being able to stand for 5 s with or without an assistive device (*p* < 0.05), and a higher frequency of family visits (*p* < 0.05).

### 3.4. Outcome Analysis

Table 5 shows the changes in health outcomes of the 210 completion participants between the pre-and post-test compared using the paired *t*-test. There was a significant improvement in the scores of the Barthel index (*p* < 0.001), IADLs (*p* < 0.01), VAS (*p* < 0.001), ISSI (*p* < 0.001), and GDS-15 (*p* < 0.01), while the utility of quality of life and MMSE score were maintained.

The main improvement in the social support domain was in emotional and information support. Each of the 10 activities in the Barthel index improved except for feeding and toilet use (*p* > 0.05).

### 3.5. Factors Affecting Effectiveness

Factors such as demographic variables, recruitment conditions (being unable to move around without a wheelchair), initial cognitive status, time of pre-test and post-test, family visit frequency, and social support from facility staff (ISSI) were included in the mixed-effect model to analyze the key factors influencing outcomes. The results are shown in Table 6. The effectiveness of ADLs was influenced by recruitment conditions (β = −17.81, *p* < 0.001), initial cognitive status (Mild cognitive impairment, β = 4.56, *p* < 0.05; Normal, β = 8.02, *p* < 0.05, compared with Severe cognitive impairment), and time (β = 4.93, *p* < 0.001). The higher the level of initial cognitive status was, the higher the ADL score was. IADLs were positively related to initial cognitive status (Mild cognitive impairment, β = 0.37, *p* < 0.01; Normal, β = 1.99, *p* < 0.001) and time (β = 0.23, *p* < 0.01), and negatively related to recruitment conditions (β = −0.84, *p* < 0.001). The utility of quality of life was positively related to initial cognitive status (Normal, β = 0.07, *p* < 0.05) and support from facility staff (β = 0.01, *p* < 0.01), and negatively related to recruitment conditions (β = −0.09, *p* < 0.001). VAS was positively related to time (β = 8.61, *p* < 0.001) and support from facility staff (β = 0.65, *p* < 0.05), and negatively related to recruitment conditions (β = −5.53, *p* < 0.05). GDS-15 was positively related to time (β = −0.59, *p* < 0.01) and support from facility staff (β = −0.23, *p* < 0.001). Furthermore, in the model of MMSE, considering that the initial cognitive status had high correlation to MMSE outcome, we removed the initial cognitive status factor in this model when analyzed; the results showed that the MMSE score was not significantly influenced by factors in the model.

## 4. Discussion

### 4.1. Characteristics of Participants in Restorative Care

Compared with previous research on the status of residents in LTC facilities in Taiwan, the average age of 81.27 years in this study was about the same, but there were more participants over the age of 85 (37.4%) in the restorative care program [28,29,30]. In this study, the participants in restorative care were mainly those who had severe dependency (21–60) (49.68%). Although participants in restorative care also had more severe depression tendency and cognitive impairment (Table 2), a few studies showed that intervention was feasible for those who scored less than 15 points in MMSE [31,32].

In consideration of the number of participants who dropped out (Table 4) and the relative short time they stayed in the program, it seems that even facility staff tends to see low function residents as a potential beneficiary at the beginning. However, restorative care seemed to fit less for those having worse ADL ability, quality of life, social support and depression status. In the comparison between dropout and completion group, we did find that those with a lower Barthel index score and with depressive symptoms tended to leave the program. A higher percentage of those who were able to stand for 5 seconds with or without an assistive device were found in the complete group. Since the implementation of restorative care could involve a higher level of physical activity, which had been proved to have no association with adverse events [19,33,34], those who were involved, however, might still worry about the higher risk of falling as a consequence, and falling was the main incident among litigation against care facilities in Taiwan [35]. The cognitive status did not show significant differences, indicating that this character may not affect the possibility of staying in the program. Furthermore, those participants with higher ISSI scores including social integration and social support and with higher EQ-5D scores tended to stay in the completion group, which also showed that residents who felt a poorer sense of social support were more likely to drop out at the baseline.

Overall, this study found that residents who seemed to have potential participating and remaining in interventions had similar levels of physical and psychological functions to those of residents in LTC facilities, even those intubated or constrained, often indicated difficultly in caring, were considered as participants at first. However, those participants with vulnerable functions and who sensed poorer support tended to be the dropouts. More information in terms of the pros and cons of restorative care seems to be critical for those involved in the program.

### 4.2. Intervention Mode and Contents

According to the literature, usual care in LTC facilities is characterized by monitoring and physical task orientation, such as the unreasonable use of physical restraints or ignoring residents’ sentiment needs while completing basic activities of daily living for them. This fails to promote residents’ autonomy and self-esteem [36]. In Taiwan, the government has made regulations for the quality of institutional care, including indicators such as the rate of restraint. However, the actual situation differs between facilities due to differences in manpower and work efficiency.

Previous research has shown that nursing homes in America have implemented restorative care programs to meet mandates and receive additional reimbursement [9,37]. A survey in the US showed that the most common restorative care activities were walking, passive and active range of motion, and dressing/grooming [9], which were primarily focused on physical and daily function; engagement with social and recreational activities to improve quality of life has not been emphasized [38].

In this study, we compiled 16 items of strategies from staff recorded in intervention notes. The items physical activities, self-care activities, assistive device, diet/water, and excretion-related items are similar to those in previous studies and were the most commonly used. Pain management, fall prevention, unconstrained, and sleep adjustment are seldom used in the literature. In addition, capacity items such as meal type recorded in the intervention note were unique observation indicators in this program compared with those used in other countries. Therefore, the aims to solve problems and the strategy used accordingly in the facilities of Taiwan may not be exactly the same as other restorative care models, leading to significant differences in the ranking of intervention items between Taiwan and other countries.

The literature from Western countries shows that the models usually target self-care abilities at the outset; however, the results in this study show that the Taiwanese model prefers to improve the overall physical and mental functions first; the initial strategies were mainly based on physical activities and supplemented by diet/water and excretion-related strategies. In the subsequent process of achieving the goal of eliminating diapers and constraint, all functional abilities including social interaction are gradually integrated for performing ADLs.

### 4.3. Effectiveness of Restorative Care

Previous research showed that restorative care in LTC facilities could slow down functional decline and increase the time and number of residents participating in physical activities, improve the performance of basic ADLs and walking ability, and decrease anxiety and agitation behavior. Other physical and psychological indicators such as SE, OEs, depression, quality of life, gait, falls, eating, and dressing are maintained after a period of at least 6 months. The long-term effects of restorative care after intervention still need further exploration [18,32,39]. In this study, after six months of intervention, significant improvements were shown in ADLs, IADLs, EQ-5D VAS, and GDS-15, while the utility of quality of life and MMSE score were maintained. These results are consistent with previous findings. However, the progress in the score of IADLs and GDS-15, as well as the maintenance of the overall quality of life (utility) are inconsistent with previous studies [5,7,37,40]. The significant improvement in the function of IADLs in this study may be due to poor function at the baseline, making it easy to reach statistical significance with little improvement (Table 5). Other discrepancies may have been caused by the differences in implementation models from country to country. When comparing the outcome changes in the same indicators to usual residential care reported in previous research [28,41,42,43], the progress or maintenance shown in our findings suggest that the restorative care program in LTC facilities in Taiwan seems to be promising. It is worthwhile to further investigate this by a case-control study to confirm the outcomes.

### 4.4. Impact of Family Visits

Although many families were willing to try restorative care, the frequency of family visits was found to be a key factor between the completion group and the dropout group. The dropout group had a significantly higher frequency of family visits.

Generally, the more family support is given, the more motivated and more satisfied residents are. However, things might be different due to filial piety in Eastern culture. Residential care tends to be the last choice for informal caregivers in Taiwan when care burden exceeds their competence. The combination of the need for skilled care and their guilt feeling may turn into a high requirement for the quality of care provided by facility staff [44,45]. Since the possible worry may still exist, this concern could be an obstacle to the program. Additionally, residents who felt a poorer sense of social support from facility staff (shown in Table 4) would also drop out at the baseline. Taking these into consideration, the results may be partially explained by insufficient communication between staff, residents, and family, as indicated in a previous study [37].

To minimize the possibility of misunderstanding, the strategy of treating family members as part of the community rather than visitors can contribute to stabilizing residents in the whole program has been suggested [38]. Also, in goal-oriented intervention, such as restorative care, the involvement of informal caregivers can help to set a realistic and achievable goal [46]. The benefits of better interaction with family by creating a coordinated social environment to encourage family participation and involvement is also found in many studies [47,48,49]. Therefore, it may be necessary to strengthen the description of knowledge among restorative care and its difference with usual care for residents and their family, and alter the care only when a consensus is reached, to avoid misunderstanding and residents’ potential from quitting the program due to doubt and worry from families.

### 4.5. Importance of Social Support in Restorative Care

Among factors affecting the effectiveness of physiological and psychological outcomes, the social support (ISSI) from facility staff was the most significant, especially in terms of quality of life and depression; it seems that the positive outcomes were related to the changes in social support, which is consistent with the social ecological model [5] to illustrate restorative care. Since this kind of philosophy requires facility staff to alter the way they treat and care for residents, we must emphasize the interpersonal level in the whole model, including the role of facility staff and residents, and the interactions between them, thus making the social support from staff a crucial factor and proving that the social ecological model could be used as the basis for verification of this study.

Previous studies have shown that front-line staff are usually willing to learn this new philosophy of care and change their beliefs and behaviors and increase the number of restorative care strategies provided to residents [31]. In addition to considering the program acceptable, staff have indicated that such interventions could help them build a better relationship with residents at the individual level through taking time to comprehend residents’ needs [38].

Regarding the domains of the social support scale (ISSI) in this study, the most significant changes in residents finishing the intervention program were emotional support and information support. It is very likely that it was the substantial concern and support during the intervention process that contributed to better health outcomes. Therefore, we should educate staff on communication and support staff before intervention is carried out, especially in terms of emotional and information provision to enhance the intervention benefit.

### 4.6. Limitations

This study is limited by the use of secondary data analyses with missing data. The power of the results is also limited given the lack of a control group due to difficulties in the implementation of collecting same data for those with usual care in LTC facilities. Future research with a rigorous experimental design is needed to confirm the effect of restorative care and examine the utility of this philosophy to care across a larger group of residents and facilities.

## 5. Conclusions

This study observed how restorative care is implemented in LTC facilities in Taiwan and explored its effectiveness. The findings showed that residents with severe dependency have great potential to participate in this program. This study confirms that restorative care may help prevent some of the persistent functional declines commonly noted among residents in LTC facilities. Also, it is critical that families are well informed and that staff involved in this program need to obtain enough information and knowledge. It is suggested that facilities need to focus on these significant factors strategically when implementing restorative care in order to allow frontline staff to change the philosophy of care, respect the human rights of residents with disabilities, and thus produce better outcomes.

## Figures and Tables

**Table 1:** Operation definitions of variables.

Variable	Coding	Operation Definition
Recruit condition—being unable to move around without a wheelchair (2 categories)	0. No (Reference)	Mainly walk with canes or other assistive advice, including total independence.
	1. Yes	Resident could only move around with wheelchairs.
Initial cognition status (3 categories)	0. Severe cognitive impairment (Reference)	0 ≤ Mini-Mental State Examination (MMSE) ≤ 15.
	1. Mild cognitive impairment	16 ≤ MMSE ≤ 23.
	2. Normal	24 ≤ MMSE ≤ 30.
Health status (6 categories)	0. Others (Reference)	Other/unspecified condition.
	1. Stroke	Includes every types of strokes.
	2. Neurological condition excluded strokes	The most common diseases in this category are Parkinson’s and Alzheimer’s disease.
	3. Arthritis	Includes every types of arthritis.
	4. Fracture	Fracture within a year.
	5. Cardiopulmonary disease	A range of serious disorders that affect the heart and lungs, e.g., Cardiovascular Disease (CVD) and Chronic Obstructive Pulmonary Disorder (COPD).
Family visit frequency (3 categories)	0. Low (Reference)	Less than 3 months.
	1. Moderate	Per month.
	2. High	Per week.
Intervention type (2 categories)	0. Combine into routine (Reference)	Integrate restorative care training strategies into resident’s original daily routine.
	1. Extra exercises	Carry out individualized training such as exercise modified for residents in a specific time. If the intervention strategies include both conditions, it would be classified as extra exercises.
Time	0. T0 (Reference)	Baseline.
	1. T1	Post-test after 6 months.
Gender	0. Male (Reference)	Male.
	1. Female	Female.
Age	Continuous variable	Age recorded at baseline (T0).
Social support from facility staff (ISSI)	Continuous variable	The total score of ISSI measured at baseline (T0).

**Table 2:** Demographic and health characteristics of residents enrolled in a trial of restorative care intervention.

Variables		T0 (*n* = 310)		Missing (*n*)
		Mean (SD)	min–max	
Age		81.27 (7.44)	65–100	
		n	%	
Sex	Male	126	40.6	
Health status		n	%	
Neurological condition excluded strokes		80	26	
Stroke		61	20	
Fracture		44	14	
Cardiopulmonary disease		44	14	
Arthritis		9	3	
others		72	24	
Family visit frequency		n	%	18
High		124	42.5	
Moderate		111	38	
Low		57	19.5	
Recruit condition (Yes)		n	%	
Being unable to move around without a wheelchair		196	63.4	1
Being able to stand for 5 seconds with or without assistive advice		239	77.3	1
Using diapers,		229	74.4	2
Being constrained		40	12.9	
Being intubated with endotracheal tube/ Foley catheter/ nasogastric (NG) tube		19	6.1	
		Mean (SD)	min–max	
ADLs		50.74 (25.32)	0–100	
IADL		0.75 (1.22)	0–6	2
EQ5D Utility		0.75 (0.44)	0–1	9
VAS		59.32 (20.95)	0–100	10
ISSI		24.66 (4.64)	11–30	18
emotional support		7.33 (1.65)	3–9	16
social integration		7.43 (1.63)	3–9	16
information support		5.08 (1.09)	2–6	17
substantive support		4.8 (1.17)	2–6	18
GDS-15		5.03 (3.93)	0–15	15
MMSE		15.56 (5.89)	0–30	19

ADLs (Activities of Daily Living); IADLs (Instrumental Activities of Daily Living); MMSE (Mini-Mental State Examination); GDS-15 (Geriatric Depression Scale-15); EQ-5D (EuroQol-5D); VAS (the Visual Analogue Scale); ISSI (Inventory of Social Support Index); ADLs category: 100 complete independence, 91–99 mild dependency, 61–90 moderate dependency, 21–60 severe dependency, and 0–20 total dependency.

**Table 3:** The changes in intervention note items.

		IN 1 ^1^		IN 2		IN 3		IN 4		
		*n* = 310		*n* = 280		*n* = 250		*n* = 222		
		n	%	n	%	n	%	n	%	*p*-Value
Seat situation When dining	No	21	6.77	15	5.36	14	5.6	15	6.76	0.004 **
	In wheelchair	211	68.06	166	59.29	136	54.4	118	53.15	
	In chair	78	25.16	99	35.36	100	40	89	40.09	
Constipation	No	188	60.7	175	62.5	172	68.8	153	68.9	0.09 *
Not using diapers		173	55.8	179	63.93	168	67.2	150	67.57	0.012 *
Not being constrained		266	85.8	242	86.43	221	88.4	199	89.64	0.53
		mean	SD	mean	SD	mean	SD	mean	SD	p-Value
Water intake		1421.72	371.71	1472.39	316.16	1577	338.3	1583.26	336.21	0 ***
Dietary intake		1479.77	158.64	1485.69	160.16	1491	164.9	1497.17	164.84	0.013 **

^1^ IN: Intervention note. * *p* < 0.05; ** *p* < 0.01; *** *p* < 0.001.

**Table 4:** Demographic characteristics and health status at baseline between completion and drop out groups.

		Completion (*n* = 210)		Drop out (*n* = 100)		
		Mean/n	SD/%	Mean/n	SD/%	*p*-Value
Age		81.74	−7.768	81.2	−6.738	0.549
Gender	Male	86	41	40	40	0.873
Health condition	Others	50	24	22	22	0.646
	Neurological condition excluding stroke	56	27	24	24	
	Stroke	39	19	22	22	
	Arthritis	8	4	1	1	
	Fracture	30	14	14	14	
	Cardiopulmonary disease	27	13	17	17	
Recruitment condition	Unable to move around without a wheelchair	129	62	67	67	0.367
	Able to stand for 5 s with or without assistive device	169	81	70	70	0.033 *
	Uses diapers	150	72	79	80	0.132
	Constrained	22	10	18	18	0.065
	Intubated with endotracheal tube/Foley catheter/nasogastric tube	10	5	9	9	0.146
Family visit frequency	High	74	35	50	50	0.028 *
	Moderate	78	37	33	33	
	Low	58	28	17	17	
ADL	Total score	53	24.349	46	26.761	0.023 *
IADL	Total score	0.83	1.3	0.56	1	0.068
GDS-15	Total score	4.24	3.51	6.79	4.25	0 ***
MMSE	Total score	15.62	5.93	15.43	5.78	0.8
EQ-5D	Utility (0–1)	0.65	0.17	0.58	0.21	0.004 **
	VAS (0–100)	61.69	19.88	54.51	22.3	0.005 **
ISSI	Total score	25.51	4.424	22.95	4.622	0.000 ***
	Emotional support	7.54	1.624	6.90	1.636	0.002 **
	Social integration	7.71	1.547	6.86	1.646	0.000 ***
	Information support	5.18	1.069	4.88	1.111	0.025 *
	Substantive support	5.05	1.042	4.32	1.263	0.000 ***

* *p* < 0.05; ** *p* < 0.01; *** *p* < 0.001. For abbreviations see Table 2.

**Table 5:** Outcome changes in the pre-test (T0) and post-test (T1).

		T0		T1		
		Mean	SD	Mean	SD	*p* Value
ADLs (*n* = 210)	Total scores	53.1	24.25	58.12	26.56	0.000 ***
	Feeding	8.79	2.556	8.76	2.748	0.882
	Dressing	5.76	3.302	6.33	3.407	0.001 **
	Toilet use	6.33	2.997	6.57	3.193	0.123
	Bathing	0.57	1.595	1.14	2.105	0.000 ***
	Transfers	9.24	5.459	9.93	5.279	0.006 **
	Mobility	7.6	4.993	8.5	5.269	0.002 **
	Stairs	1.45	2.751	1.95	3.169	0.006 **
	Grooming	3.45	2.317	3.76	2.163	0.012 *
	Bowels	2.4	2.242	5.67	3.761	0.000 ***
	Bladder	4.71	3.809	2.33	2.199	0.000 ***
IADLs (*n* = 210)	Total scores	0.83	1.3	1.06	1.5	0.001 **
EQ-5D	Utility (*n* = 202)	0.65	0.17	0.66	0.18	0.161
	VAS (*n* = 200)	61.43	19.83	70.1	17.95	0.000 ***
ISSI (*n* = 193)	Total score	25.5	4.45	26.49	3.94	0.000 ***
	Emotional support	7.53	1.635	8.01	1.42	0.000 ***
	Social integration	7.7	1.55	7.9	1.43	0.054
	Information support	5.2	1.06	5.45	0.86	0.001 **
	Substantive support	5.03	1.04	5.1	1.02	0.294
GDS-15 (*n* = 198)		4.24	3.51	3.61	2.9	0.001 **
MMSE (*n* = 195)		15.75	5.91	15.87	6.04	0.698

*p < 0.05; **p < 0.01; ***p < 0.001. For abbreviations see Table 2.

**Table 6:** Influencing factors of health outcomes in mixed-effect models.

*N* = 310	ADLs	IADLs	EQ-5D Utility	EQ-VAS	GDS-15	MMSE
	Estimated mean difference (Std. Error)					
Fixed effects						
Gender	4.32 (3.31)	−0.06 (0.16)	0.01 (0.02)	−4.34 (2.27)	0.11 (0.41)	−1.64 (0.83)
Age	0.30 (0.22)	0.00 (0.01)	0.00 (0.00)	0.13 (0.15)	−0.01 (0.03)	−0.05 (0.05)
Time (ref. “T0”)	4.93 (1.12) ***	0.23 (0.07) **	0.02 (0.01)	8.61 (1.57) ***	−0.59 (0.19) **	0.17 (0.33)
Being unable to move around without a wheelchair (ref. “no”)	−17.81 (3.32) ***	−0.84 (0.17) ***	−0.09 (0.02) ***	−5.53 (2.25) *	0.75 (0.41)	0.25 (0.80)
Cognitive function (ref. “Severe”)						
Mild cognitive impairment	4.56 (2.09) *	0.37 (0.12) **	0.02 (0.02)	1.32 (2.08)	0.18 (0.31)	
Normal	8.02 (3.27) *	1.99 (0.19) ***	0.07 (0.03) *	5.87 (3.16)	−0.27 (0.50)	
Diseases (ref. “others”)						
Stroke	−2.72 (4.83)	−0.10 (0.23)	0.01 (0.03)	−1.27 (3.28)	0.85 (0.58)	−1.45 (1.23)
Neurological condition excluding stroke	−2.80 (4.25)	−0.22 (0.20)	0.02 (0.03)	3.79 (2.91)	0.28 (0.51)	−0.52 (1.09)
Arthritis	4.54 (8.25)	0.06 (0.39)	−0.01 (0.06)	0.86 (5.53)	0.73 (0.98)	1.27 (2.11)
Fracture	0.76 (5.16)	0.13 (0.25)	0.04 (0.04)	−0.05 (3.49)	1.06 (0.62)	−0.77 (1.33)
Cardiopulmonary disease	3.89 (5.27)	−0.15 (0.25)	0.04 (0.04)	5.89 (3.62)	0.36 (0.64)	1.61 (1.35)
Family visit frequency (ref. “Low”)						
Moderate	−4.31 (3.80)	0.04 (0.19)	−0.03 (0.03)	−1.00 (2.78)	−0.28 (0.49)	−0.90 (0.98)
High	−4.94 (3.68)	0.16 (0.18)	−0.05 (0.03)	−2.49 (2.76)	0.50 (0.48)	−1.14 (0.96)
ISSI	0.37 (0.39)	0.01 (0.02)	0.01 (0.00) **	0.65 (0.26) *	−0.23 (0.05) ***	0.00 (0.09)
Intervention type: Extra exercises (ref. “Combine into routine”)	4.77 (4.97)	−0.53 (0.36)	−0.02 (0.02)	−2.67 (2.99)	0.82 (0.69)	−0.45 (0.88)
Random effects						
Facilities	58.26	0.46	0.00	16.16	1.35	0.01

* *p* < 0.05; ** *p* < 0.01; *** *p* < 0.001. For abbreviations see Table 2.
